# Exploring Novel Molecular Targets for Alzheimer’s Disease in Neuropharmacology

**DOI:** 10.1002/alz.085882

**Published:** 2025-01-09

**Authors:** Shampa Ghosh, Krishna Kumar Singh, Aparajita Dasgupta Amist, Jitendra Kumar Sinha

**Affiliations:** ^1^ ICMR ‐ National Institute of Nutrition, Hyderabad, Telangana India; ^2^ GloNeuro Academy, Noida, Uttar Pradesh India; ^3^ Symbiosis Centre for Information Technology, Pune, Maharastra India; ^4^ Amity University, Noida, Uttar Pradesh India

## Abstract

**Background:**

Alzheimer’s disease (AD) remains a formidable neurodegenerative challenge, characterized by profound cognitive decline. Despite decades of research, effective disease‐modifying therapies are elusive. Recent advances in molecular neuropharmacology have unveiled potential therapeutic targets for AD, offering renewed hope.

**Method:**

This comprehensive review explores emerging avenues to combat AD, emphasizing diverse targets with substantial promise. Key focus areas include beta‐secretase (BACE) and gamma‐secretase, crucial in amyloid‐beta (Aβ) peptide formation—the hallmark of AD pathology. The review also dissects tau protein abnormalities, neuroinflammation cascades, and proteins associated with synaptic dysfunction. Innovative modalities, such as immunotherapies and microRNA‐based interventions, are discussed.

**Result:**

Novel therapeutic targets introduce groundbreaking dimensions to AD therapeutics, transcending traditional approaches. Immunotherapies and gene regulation techniques aim not only for symptomatic relief but also to arrest disease progression, enhancing overall patient and caregiver well‐being.

**Conclusion:**

Understanding these emerging targets, coupled with delving into their underlying mechanisms, signifies a paradigm shift in AD intervention. The goal is not merely symptomatic alleviation but halting disease advancement. However, translating these promising prospects into clinical reality demands unwavering commitment to rigorous scientific scrutiny and meticulous clinical validation. Systematic trials are imperative to establish the efficacy, safety, and long‐term benefits of these potential treatments, ensuring their transformative impact on AD care.

